# Fine-Tuned Bidirectional Encoder Representations From Transformers Versus ChatGPT for Text-Based Outpatient Department Recommendation: Comparative Study

**DOI:** 10.2196/47814

**Published:** 2024-10-18

**Authors:** Eunbeen Jo, Hakje Yoo, Jong-Ho Kim, Young-Min Kim, Sanghoun Song, Hyung Joon Joo

**Affiliations:** 1 Department of Medical Informatics Korea University College of Medicine Seoul Republic of Korea; 2 Department of Bio-Mechatronic Engineering Sungkyunkwan University College of Biotechnology and Bioengineering Gyeonggi Republic of Korea; 3 Medical AI Research Center, Research Institute for Future Medicine Samsung Medical Center Seoul Republic of Korea; 4 Korea University Research Institute for Medical Bigdata Science Korea University Seoul Republic of Korea; 5 Department of Cardiology, Cardiovascular Center Korea University College of Medicine Seoul Republic of Korea; 6 School of Interdisciplinary Industrial Studies Hanyang University Seoul Republic of Korea; 7 Department of Linguistics Korea University Seoul Republic of Korea

**Keywords:** natural language processing, bidirectional encoder representations from transformers, large language model, generative pretrained transformer, medical specialty prediction, quality of care, health care application, ChatGPT, BERT, AI technology, conversational agent, AI, artificial intelligence, chatbot, application, health care

## Abstract

**Background:**

Patients often struggle with determining which outpatient specialist to consult based on their symptoms. Natural language processing models in health care offer the potential to assist patients in making these decisions before visiting a hospital.

**Objective:**

This study aimed to evaluate the performance of ChatGPT in recommending medical specialties for medical questions.

**Methods:**

We used a dataset of 31,482 medical questions, each answered by doctors and labeled with the appropriate medical specialty from the health consultation board of NAVER (NAVER Corp), a major Korean portal. This dataset includes 27 distinct medical specialty labels. We compared the performance of the fine-tuned Korean Medical bidirectional encoder representations from transformers (KM-BERT) and ChatGPT models by analyzing their ability to accurately recommend medical specialties. We categorized responses from ChatGPT into those matching the 27 predefined specialties and those that did not. Both models were evaluated using performance metrics of accuracy, precision, recall, and *F*_1_-score.

**Results:**

ChatGPT demonstrated an answer avoidance rate of 6.2% but provided accurate medical specialty recommendations with explanations that elucidated the underlying pathophysiology of the patient’s symptoms. It achieved an accuracy of 0.939, precision of 0.219, recall of 0.168, and an *F*_1_-score of 0.134. In contrast, the KM-BERT model, fine-tuned for the same task, outperformed ChatGPT with an accuracy of 0.977, precision of 0.570, recall of 0.652, and an *F*_1_-score of 0.587.

**Conclusions:**

Although ChatGPT did not surpass the fine-tuned KM-BERT model in recommending the correct medical specialties, it showcased notable advantages as a conversational artificial intelligence model. By providing detailed, contextually appropriate explanations, ChatGPT has the potential to significantly enhance patient comprehension of medical information, thereby improving the medical referral process.

## Introduction

Natural language processing technology has the potential to transform the process of health care and further improve the quality of care [1]. Among natural language processing deep learning models, transformer-based models, including bidirectional encoder representations from transformers (BERT), GPT, and XLNet, have shown excellent performance in many health care applications, such as clinical coding [2], named entity recognition [3], and disease prediction based on clinical notes [4]. Both BERT and GPT are advanced deep learning models that use transformer architectures, but they are fundamentally different. BERT is designed for bidirectional understanding of text, while GPT is designed for generative tasks and uses a unidirectional approach [5,6]. In particular, ChatGPT is a large language model (LLM) developed by OpenAI as an instance of GPT-3.5 that generates human-like text responses to a wide range of prompts and questions [7-9]. ChatGPT performed at or near the passing threshold of 60% accuracy on the United States Medical Licensing Examination, suggesting the potential integration into clinical decision-making [8]. Recently, the application of ChatGPT for general users seeking medical information has been highlighted [10,11].

The disparity in medical knowledge and literacy between health care professionals and the general public, often termed as information asymmetry, may inadvertently result in an inappropriate allocation of medical services due to misunderstandings or lack of awareness about health conditions [12,13]. Identifying the right outpatient specialist for their symptoms can be challenging for patients and often results in added costs and time. This is exacerbated by the current referral system, which leads to delays and increased missed clinical appointments [14,15]. Improving the process of identifying suitable medical professionals can enhance the quality of care, reduce costs, and boost patients’ satisfaction [16]. To address this issue, we developed Korean Medical BERT (KM-BERT), a medical domain–specific pretrained BERT model, which was trained on a corpus of 6 million sentences from medical textbooks, health information news, and medical research papers [17]. Furthermore, we developed the fine-tuned KM-BERT model capable of recommending medical specialties based on general user queries [18].

Comparing these models can reveal which types of tasks each model is better suited to in the health care domain. For instance, one model may excel at predicting disease outcomes based on patient notes, while the other might be better at generating human-like text for health-related chatbots. In this study, we compare the performance of this model with ChatGPT and a previously developed BERT model, in line with previous research.

## Methods

### Data Collection

The previous BERT study collected 82,312 health care counsel posts from the NAVER portal, a Korean portal that provides medical questions and answers to general users [18]. The data-set was collected from the NAVER portal, a Korean portal that provides medical questions and answers to general users. The medical question involves the portal user describing their symptoms and requesting medical advice and information, which includes laboratory tests, medications, procedures, presumptive diagnoses, and recommendations for health professionals and institutions. Medical questions posted by users of the portal are reviewed and responded to by certified doctors through the portal. Each post also includes a label indicating the relevant medical specialty. The dataset consisted of questions and medical specialty label pairs. Medical specialty labels for the questions were limited to 27 clinical departments for the development of the BERT model. The original dataset was divided into a training set consisting of 50,454 data pairs and a test set comprising 31,482 data pairs. The training set was used to develop the fine-tuned KM-BERT model through 5-fold cross-validation. From the original test set, wherein data pairs were posted between July 13, 2021, and September 13, 2021, this study used 31,482 data pairs after excluding 376 due to missing data (Table 1). The medical questions asked to ChatGPT are the same as the test set (31,482 data pairs) used to develop the fine-tuned KM-BERT model.

**Table 1:** The number of test data used to measure the performance of ChatGPT and the fine-tuned Korean Medical bidirectional encoder representations from transformers (KM-BERT) model (N=31,482).

Specialty	Value, n (%)
Anesthesiology	1980 (6.29)
Cardiac and thoracic surgery	46 (0.15)
Cardiology	184 (0.58)
Dentistry	1980 (6.29)
Dermatology	1980 (6.29)
Emergency medicine	591 (1.88)
Endocrinology	169 (0.54)
Family medicine	1980 (6.29)
Gastroenterology and hepatology	306 (0.97)
General surgery	3268 (10.38)
Hematology and oncology	156 (0.50)
Infectious diseases	146 (0.46)
Nephrology	67 (0.21)
Neurology	558 (1.77)
Neurosurgery	1980 (6.29)
Obstetrics and gynecology	2644 (8.40)
Ophthalmology	1980 (6.29)
Orthopedic surgery	1980 (6.29)
Otolaryngology	1980 (6.29)
Pediatrics	389 (1.24)
Plastic surgery	1980 (6.29)
Psychiatry	500 (1.59)
Pulmonology	43 (0.14)
Radiology	422 (1.34)
Rehabilitation medicine	1980 (6.29)
Rheumatology	213 (0.68)
Urology	1980 (6.29)

### Generating ChatGPT Medical Specialty Recommendations for Questions

ChatGPT is based on the GPT-3.5 series, and this study used “text-davinci-003” model, the latest version of the GPT-3.5 models available from the OpenAI application programming interface service at the time of the study [8,9]. ChatGPT has a better understanding of English than low-resource languages [19,20]. The questions were translated from Korean to English using the Google Translation application programming interface [21]. Previous research has also been successful in translating medical words and sentences from Korean to English [17]. ChatGPT can improve question comprehension depending on the prompting strategy [22]. To prompt ChatGPT to answer the questions, the question was appended with the sentence, “In this case, which clinical department in the hospital would be better? Please recommend 3 in order of priority.”

The training corpus used for ChatGPT has not been publicly disclosed, but it is understood that it was trained on a vast amount of text data from multiple languages and sources, including Korean [8,19,23]. However, for this study, only translated sentences were used as inputs, which means they were not part of the original training samples used to develop ChatGPT. Furthermore, the original questions were randomly cross-checked to ensure that they were not indexed on Google.

### Evaluating the Performance of KM-BERT and ChatGPT

This study was conducted in strict accordance with the “Guidelines for Developing and Reporting Machine Learning Predictive Models in Biomedical Research” as published by JMIR [24]. The performance of appropriate medical specialty recommendations for medical questions from fine-tuned KM-BERT and ChatGPT was evaluated based on the same test set and 27 medical specialty labels. A confusion matrix for the 27 specialties was created to compare the first recommendation from each model to the correct medical specialty labels and to calculate true positives, false positives, true negatives, and false negatives [25]. With an imbalance of data for each medical specialty, the performance was evaluated using macro-averaging accuracy, macro-averaging precision, macro-averaging recall, and macro-averaging *F*_1_-score. The last layer of the fine-tuned KM-BERT used the softmax activation function for multiclassification, and performance was measured by comparing the first predicted medical specialty to the correct medical specialty label. The responses from ChatGPT were categorized into those that corresponded to the 27 predefined specialties and those that did not. This categorization was necessary because ChatGPT provided some responses that did not fit within the 27 predefined specialties. Out of a total of 31,482 questions, ChatGPT supplied first-rank responses corresponding to the 27 medical specialties 29,534 times (93.8%), second-rank responses 21,191 times (67.3%), and third-rank responses 19,291 times (61.3%).

### Ethical Considerations

This research project, including the original data collection, was approved by the institutional review board of Korea University Anam Hospital (2024AN0315).

## Results

### Medical Specialty Recommendations by ChatGPT

ChatGPT was able to recommend medical specialties for 29,534 (93.8%) of the total 31,482 questions. ChatGPT declined to answer the rest of the questions (eg, “Unfortunately, I cannot answer your question as I am not a qualified medical professional and cannot provide legal advice”). The responses provided by ChatGPT covered a wide range of 1685 clinical departments, centers, clinics, hospitals, and medical specialists. However, some of the responses did not fit into the predefined 27 clinical departments, with “department of internal medicine” being a common general response. ChatGPT also provided some answers that were not classifiable, such as those relating to medical schools or hospitals that could not be categorized (eg, “Korea University College of Medicine,” “Seoul National University Bundang Hospital,” and “Johns Hopkins Hospital”). ChatGPT gave hallucinated answers relating to clinics that were not actual locations, like “K Dental Clinic” [19]. Overall, 842 of the 1685 distinct responses were able to be classified into 1 of the 27 clinical departments.

**Figure 1:**
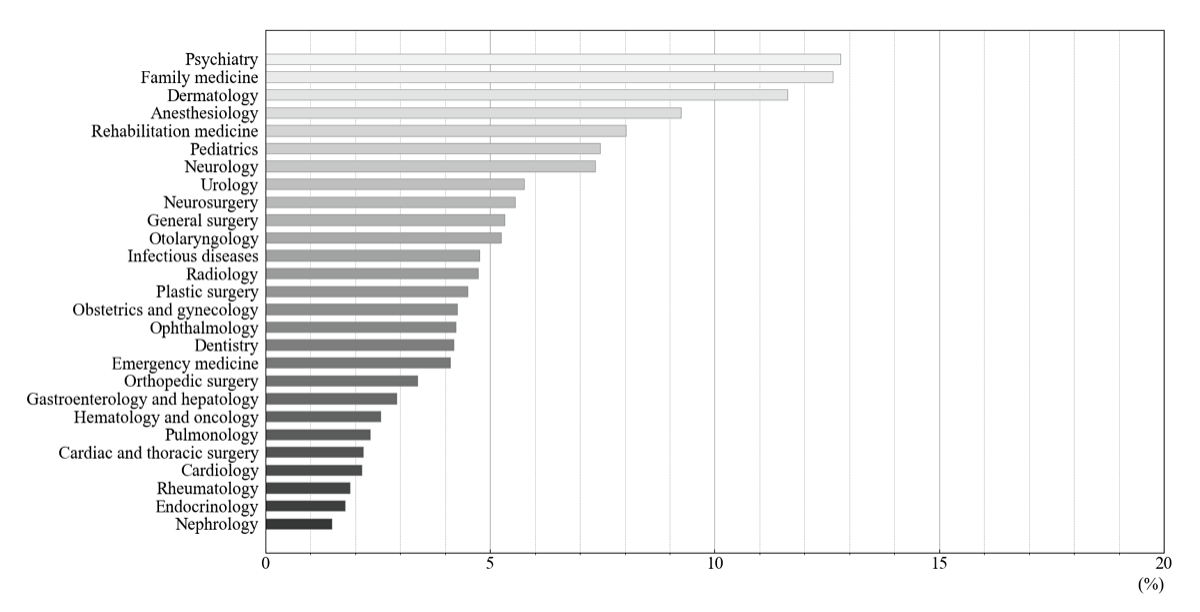
Answer avoidance rate of ChatGPT to the medical specialty recommendation.

ChatGPT had an answer avoidance rate of 6.2% for inquiries regarding medical specialty recommendations. Figure 1 illustrates the response avoidance rate for each department of ChatGPT. Psychiatry had the highest avoidance rate, followed by family medicine and dermatology. On the other hand, nephrology, endocrinology, and rheumatology had the lowest avoidance rates, in that order.

### Performance of ChatGPT and KM-BERT

**Figure 2:**
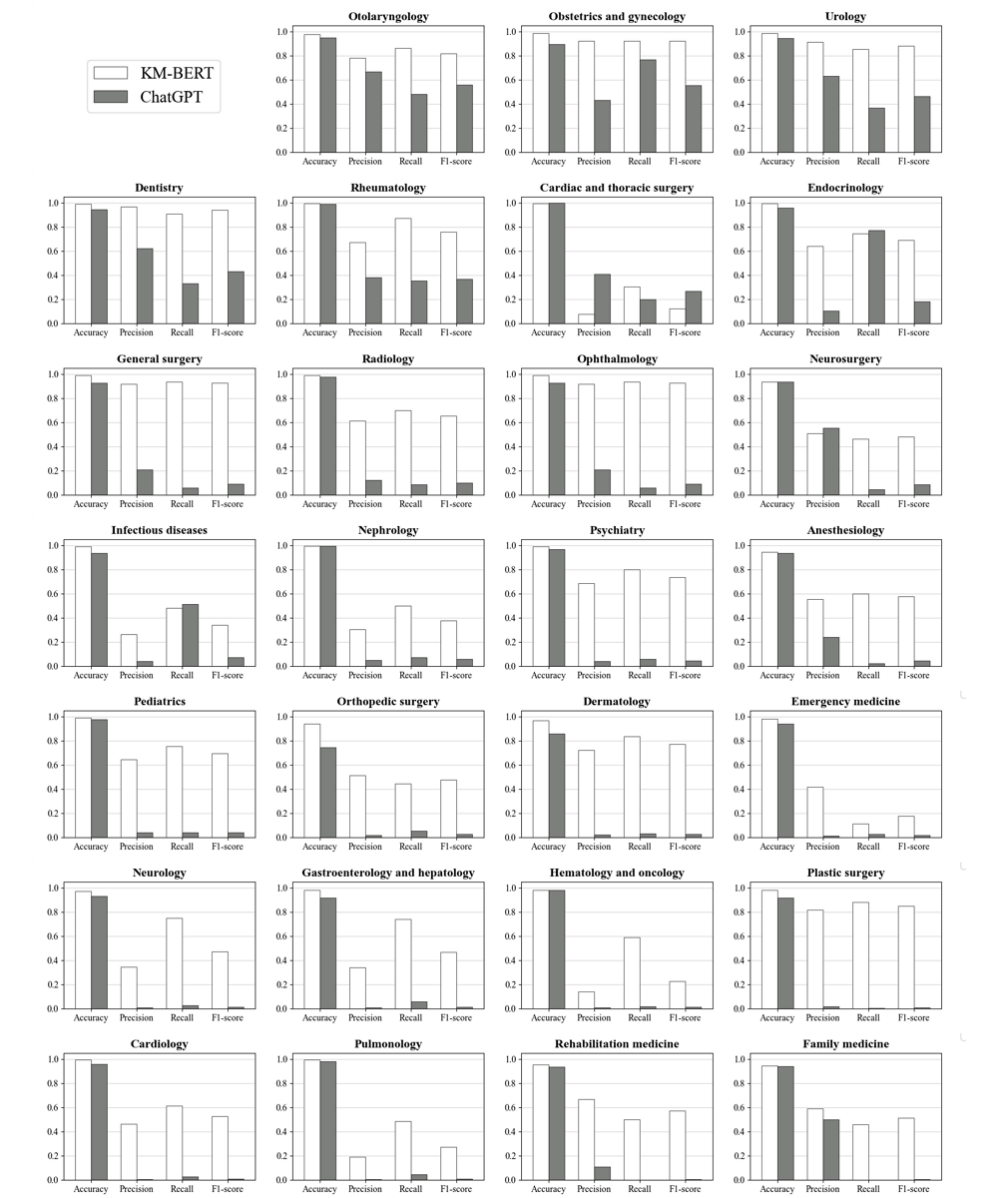
Accuracy, precision, recall, and F1-score of ChatGPT and KM-BERT for each department of test set evaluation. KM-BERT: Korean Medical bidirectional encoder representations from transformers.

ChatGPT’s overall performance on medical specialty recommendations was lower than the fine-tuned KM-BERT model (accuracy 0.939 for ChatGPT vs 0.977 for KM-BERT, precision 0.219 for ChatGPT vs 0.570 for KM-BERT, recall 0.168 for ChatGPT vs 0.652 for KM-BERT, *F*_1_-score 0.134 for ChatGPT vs 0.587 for KM-BERT). In ChatGPT, the departments with the highest *F*_1_-score were otolaryngology, obstetrics and gynecology, and urology, in that order, and the departments with the lowest *F*_1_-score were family medicine, rehabilitation medicine, and pulmonology (Figure 2).

## Discussion

### Principal Findings

**Figure 3:**
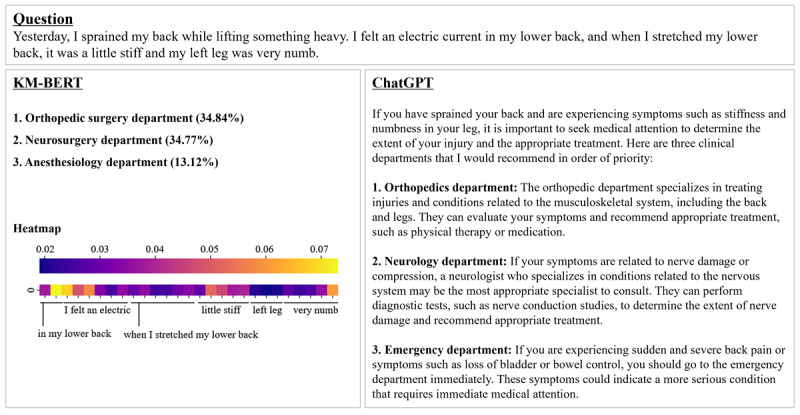
Medical specialty recommendation results of ChatGPT and BERT models. Left: output information from the KM-BERT model. The BERT model reliably predicts the medical specialty based on the calculated probability. The heatmap shows the average attention for each token, which can provide insights into the model’s decision-making process. The greater the brightness, the more attention. The order of the text under the heatmap has been changed as it was translated from Korean. Right: output from ChatGPT. Based on the input information, the model infers key pathophysiology and keywords from a medical perspective to recommend the appropriate medical specialty. BERT: bidirectional encoder representations from transformers; KM-BERT: Korean Medical bidirectional encoder representations from transformers.

In the health care industry, it is crucial to provide patients with a clear justification or explanation for any artificial intelligence (AI)–based recommendations [26,27]. The growing demand for explainable AI technology in health care is consistent with this requirement [28]. ChatGPT is a significantly more advanced model than BERT in this regard [29]. For instance, when presented with the query, “Yesterday, I sprained my back while lifting something heavy. I felt an electric current in my lower back, and when I stretched my lower back, it was a little stiff and my left leg was very numb.” While BERT can accurately suggest the most appropriate medical specialty in all cases, it can only offer a rough estimation by identifying the token the model is focusing on through a heatmap, etc (Figure 3). In contrast, ChatGPT can deduce the fundamental pathophysiology of the patient’s primary symptoms and provide a medical specialty recommendation accompanied by an explanation of the rationale, resulting in increased credibility and acceptance of the recommendation from the user’s perspective, even if it cannot address all inquiries. This may be one of the biggest advantages of ChatGPT as a conversational language model.

While ChatGPT did not outperform the fine-tuned BERT model in recommending departments for health care services, it displayed numerous advantages as a conversational language model. The advantages of ChatGPT can be useful in the health care industry. First, ChatGPT can be applied to medical consultations to help patients understand medical information. Patients prefer to receive information that is written in plain language, particularly in health care, where there is an unfamiliar amount of terminology [30]. Enhancing the ability of individuals to understand and interpret the meaning of health information needed to make appropriate health decisions can improve the efficiency of the health care system [31-33]. Second, ChatGPT can assist clinicians in evaluating and diagnosing a patient’s symptoms. Patients sometimes have difficulty describing their symptoms [34]. By analyzing patients’ textual descriptions, ChatGPT can provide a more specific description of their symptoms, which can help clinicians better understand their patients and provide appropriate treatment [35].

The relatively poor performance of ChatGPT in this exploratory study could be attributed to the fact that the data sources used for its development were general data, mainly US-based data, with relatively little medical-specific data [20]. However, OpenAI has recently launched a fine-tuning service for ChatGPT, which is expected to significantly enhance its performance. Fine-tuning will be especially crucial since each country operates a different medical service system. As a result, we can anticipate the emergence of several ChatGPT variants fine-tuned for use in the health care industry in the future.

Finally, while ChatGPT offers incredible possibilities, concerns about the potential for generating untrue statements are growing [19,36]. As a generative model, some inaccuracies are inevitable, but they can be mitigated through fine-tuning with high-quality and reliable data resources [19,37]. It is also essential to develop and implement algorithms that can fact-check ChatGPT’s statements [38]. By addressing these limitations, we can continue to explore the exciting potential of ChatGPT, ensuring that it remains a useful tool for the future of health care.

### Limitations

This study has several limitations. First, the training datasets used for the 2 models were entirely distinct. Despite the extensively large corpus upon which ChatGPT is trained, the KM-BERT model, due to its pretraining with a corpus specific to the medial domain, may exhibit superior performance in the task of medical specialty classification. Second, diverse prompting strategies can affect the classification performance of ChatGPT. A recent study revealed a comparative underperformance of contemporary LLMs against smaller, fine-tuned BERT models, particularly in a zero-shot setting [39]. Moreover, the accuracy and *F*_1_-scores of LLMs differed significantly, by upwards of 10%, contingent upon the prompting strategy that is adopted. It suggests that the application of advanced prompting methodologies, such as autogenerate prompting and chain-of-thought prompting, could potentially enhance the performance of ChatGPT in the context of this study’s task [40,41]. Third, this study provides insight into the medical inference ability of ChatGPT through the medical specialty classification and a use case scenario. However, it does not extend to a quantitative evaluation of other complementary studies through objective experimentation. Notably, this study used real-world case data, not included in ChatGPT’s training phase. The other previous study has also highlighted ChatGPT’s capability to deduce medical symptoms, diagnoses, and treatments without explicit medical training [6]. The impact of the additional inferred information generated by ChatGPT on users’ decision-making process and behavioral change necessitates further exploration.

### Conclusions

In conclusion, this study highlighted the capabilities of AI models, such as fine-tuned KM-BERT and ChatGPT, in recommending medical specialties based on general user queries. The fine-tuned KM-BERT model performed better in this task, while ChatGPT showed its strengths as a conversational AI model that can provide more context-aware responses. Future studies could aim to leverage the strengths of each model to create a more comprehensive and effective system for recommending medical specialties. This could improve the health care referral process and result in better health outcomes for patients. Moreover, with the availability of fine-tuning services for ChatGPT, we can expect the development of many more specialized AI models, potentially revolutionizing the delivery of health care information to patients.

## Abbreviations

AI: artificial intelligence
BERT: bidirectional encoder representations from transformers
KM-BERT: Korean Medical bidirectional encoder representations from transformers
LLM: large language model

## Data Availability

The data that support the findings of this study are available from the corresponding author, HJJ, upon reasonable request.
